# Scabies Mites Alter the Skin Microbiome and Promote Growth of Opportunistic Pathogens in a Porcine Model

**DOI:** 10.1371/journal.pntd.0002897

**Published:** 2014-05-29

**Authors:** Pearl M. Swe, Martha Zakrzewski, Andrew Kelly, Lutz Krause, Katja Fischer

**Affiliations:** 1 QIMR Berghofer Medical Research Institute, Infectious Diseases Program, Biology Department and Genetics and Computational Biology Department, Brisbane, Queensland, Australia; 2 Department of Agriculture, Fisheries and Forestry, Queensland Animal Science Precinct, University of Queensland, Gatton Campus, Queensland, Australia; National Institute of Allergy and Infectious Diseases, United States of America

## Abstract

**Background:**

The resident skin microbiota plays an important role in restricting pathogenic bacteria, thereby protecting the host. Scabies mites (*Sarcoptes scabiei*) are thought to promote bacterial infections by breaching the skin barrier and excreting molecules that inhibit host innate immune responses. Epidemiological studies in humans confirm increased incidence of impetigo, generally caused by *Staphylococcus aureus* and *Streptococcus pyogenes*, secondary to the epidermal infestation with the parasitic mite. It is therefore possible that mite infestation could alter the healthy skin microbiota making way for the opportunistic pathogens. A longitudinal study to test this hypothesis in humans is near impossible due to ethical reasons. In a porcine model we generated scabies infestations closely resembling the disease manifestation in humans and investigated the scabies associated changes in the skin microbiota over the course of a mite infestation.

**Methodology/Principal Findings:**

In a 21 week trial, skin scrapings were collected from pigs infected with *S. scabies* var. *suis* and scabies-free control animals. A total of 96 skin scrapings were collected before, during infection and after acaricide treatment, and analyzed by bacterial 16S rDNA tag-encoded FLX-titanium amplicon pyrosequencing. We found significant changes in the epidermal microbiota, in particular a dramatic increase in *Staphylococcus* correlating with the onset of mite infestation in animals challenged with scabies mites. This increase persisted beyond treatment from mite infection and healing of skin. Furthermore, the staphylococci population shifted from the commensal *S. hominis* on the healthy skin prior to scabies mite challenge to *S. chromogenes*, which is increasingly recognized as being pathogenic, coinciding with scabies infection in pigs. In contrast, all animals in the scabies-free cohort remained relatively free of *Staphylococcus* throughout the trial.

**Conclusions/Significance:**

This is the first experimental *in vivo* evidence supporting previous assumptions that establishment of pathogens follow scabies infection. Our findings provide an explanation for a biologically important aspect of the disease pathogenesis. The methods developed from this pig trial will serve as a guide to analyze human clinical samples. Studies building on this will offer implications for development of novel intervention strategies against the mites and the secondary infections.

## Author Summary

Scabies is a neglected, contagious skin disease caused by a parasitic mite *Sarcoptes scabiei*. It is highly prevalent world-wide, and now recognized as a possible underlying factor for secondary bacterial infections with potential serious downstream complications. There is currently few experimental data demonstrating directly that mite infestation promotes bacterial infections. Due to remarkable similarities in terms of immunology, physiology and skin anatomy between pigs and humans, we developed a sustainable porcine model enabling *in vivo* studies of scabies mite infestations. Here, we investigated the impact of the scabies mite infection on the normal pig skin microbiota in the inner ear pinnae in young piglets. Samples obtained prior to, during infection and after acaricide treatment were analyzed by sequencing of bacterial 16S rDNA. We report that scabies infestation has an impact on the host's skin microbiota. *Staphylococcus* abundance increased with the onset of infection and remained beyond treatment and healing. A shift from commensal to pathogenic *Staphylococci* was observed. This study supports the link between scabies and Staphylococcus infections, as seen in humans. It is the first *in vivo* demonstration of a mite induced shift in the skin microbiota, providing a basis for a similar study in humans.

## Introduction

Scabies is a skin disease caused by the parasitic mite *Sarcoptes scabiei* variety *hominis* in humans. It is common worldwide, predominantly affecting overcrowded, socio-economically disadvantaged populations [1]. Scabies is ubiquitous and a significant public health burden in the developing world with prevalence of up to 70% in rural India and between 18 and 42% in the South Pacific, and erratic reports from Africa and South America [2], [3]. Recently scabies outbreaks are also reported regularly in economically rich regions, particularly from institutional settings such as health care facilities, elderly homes, prisons and child care centers [4], [5], where it is a well recognized and serious problem and control is notoriously difficult. Some studies suggest that the general population in economically stable societies has recently become more affected [6]. Moreover, scabies is a significant public health burden in the Indigenous population of tropical northern Australia [7]–[9]. Crusted scabies, a highly contagious manifestation of the disease presenting with extreme parasite numbers, can be seen predominantly in elderly individuals or in immunocompromised patients, especially those with infections due to HIV and human T lymphotropic virus 1, or drug-induced immunosuppression [10].

Epidemiological studies indicated a link between the epidermal infestation with *S. scabiei* and cutaneous bacterial infections (pyoderma, impetigo), particularly in tropical settings [9], [11]–[14]. Secondary bacterial skin infections commonly associated with scabies infestations are primarily caused by two clinically important pathogens, i.e. *Streptococcus pyogenes* and *Staphylococcus aureus*, including methicillin-resistant and methicillin-sensitive strains [8], [15]. These bacterial pathogens potentially cause life-threatening invasive infections, if left untreated. One obvious factor for the close association between mite infestation and bacterial disease is the breach of the physical barrier, i.e. the epidermal layers of the intact skin, through mechanical infringement by the burrowing scabies mites. This creates a suitable niche for pathogens to establish in the mite burrows. Our recent studies showed that scabies mites interfere locally with human complement mediated protection [16], [17], thereby promoting growth of *S. pyogenes* [18] and *S. aureus* (Swe *et al*., manuscript in preparation). However, the tripartite interactions between host, mites and bacteria are largely unexplored. In particular it is unknown whether scabies mites facilitate the transmission of pathogens or rely on obligatory endosymbionts for survival. Also, scabies-induced changes in the skin microbiome could provide a surrogate diagnostic biomarker for ordinary scabies infections, which are notoriously difficult to diagnose by conventional methods. Investigations into the identification of all bacteria associated with scabies mite infestation are important in developing novel control strategies as well as in improving current management and prevention policies.

Obtaining clinical samples from human scabies patients is generally a logistical challenge, as scabies is not a notifiable disease, it is difficult to diagnose and outbreaks are sporadic. Ordinary scabies patients harbor only few mites, which complicates targeted sampling. In humans, scabies manifests at multiple skin sites providing multiple, highly diverse habitats for bacteria [19]. Additional elements such as climate, hygienic procedures, age and genetics potentially increase the variation of mite associated microbiota further. Finally, a study in human patients over the course of an infection, in line with ethical limitations, would be very difficult. Therefore a longitudinal pilot study of a defined infection site in a controlled porcine animal model was undertaken. Pigs are natural hosts for *S. scabiei* var. *suis*, developing clinical manifestations closely resembling human scabies [20]. The integumentary system, the innate immunity and many biochemical parameters are incredibly similar between pigs and humans [21]–[26] making the pig a well-recognized model to study human infectious diseases [27].

Focusing on the natural site of primary mite infestations in young pigs, i.e. the inner surface of the ear pinnae, we investigated the impact of a *S. scabiei* var. *suis* infection on the skin microbiota. In a longitudinal study conducted over 21 weeks we compared the 16S rRNA gene sequences reflecting the development of the normal skin microbial community structure in healthy control animals with the microbiota present prior to, during and after *S. scabiei* var. *suis* infection.

## Methods

### Ethics statement

Animal care and handling procedures used in this study followed the Animal Care and Protection Act, in compliance with the Australian code of practice for the care and use of animals for scientific purposes, outlined by the Australian National Health and Medical Research Council. The study was approved by the Centre for Advanced Animal Science (CAAS) and the QIMR Berghofer Medical Research Institute Animal Ethics Committees (DEEDI-AEC SA2012/02/381, QIMR A0306-621M).

### Animals

Pigs were housed at the CAAS, Gatton, QLD. A total of 15 female, 3 weeks old *Sus scrofa domesticus* “Large White” breed siblings from the same pig breeding facility were adjusted to standard stable and feed conditions for 3 weeks prior to the start of the trial. After one week pigs were allocated randomly to 3 experimental groups (n = 5) and housed in identical but separate standardized, climate-controlled rooms, set at an average temperature of 27°C–30°C. All rooms were run on a continuous flow basis and concrete floors were cleaned twice daily. To assist monitoring and sampling, the formation of skin lesions and crusts associated with mite infestation was recorded on a weekly basis using an established scoring system [28].

### Trial design

The tractable experimental porcine scabies model was developed previously [28] where high mite numbers are observed in the inner surface of the ear pinnae of young piglets. The development of crusted scabies is generally seen within 10 to 13 weeks after mite infestation, but only in a subset of infected animals. The majority of individuals, analogously to humans, develops ordinary scabies and then, depending on the mite challenge, overcomes the infection without developing severe infestation and crust formation. Therefore, a previously developed strategy [28] was adopted for one cohort of the trial, where extreme parasite infestation was achieved by treatment with the synthetic gluco-corticoids immune-suppressant Dexamethasone (Provet, Brisbane). Synthetic gluco-corticoids are commonly used to promote infection in animal models [29], and the crusted scabies following corticosteroid therapy has been observed in humans [30]. The schedule of 1 week as adjustment period to stable conditions and of subsequent 2 weeks as the period to achieve in all individuals the Dexamethasone levels required for fast mite infestation was previously established [28].

The three experimental cohorts of pigs served as a Mite infected group (M), a Mite infected and Dexamethasone treated group (MD), and an uninfected, untreated Control group (C) (Figure S1, supplemental information). The MD group was administered a daily oral dose of 0.2 mg Dexamethasone per kg of body weight, starting 2 weeks prior to scabies mite challenge and sustained continuously throughout the trial (week -2). At the start of the trial (week 0), prior to infection of the cohorts M and MD, a scraping of approximately 1×1 cm^2^ of the epidermal layer of the skin was sampled with a sterile curette (Ø = 7 mm, Stiefel Laboratories Pty Ltd). This baseline sample was taken from the same site on the inside of the right ear pinna of each pig across all cohorts. Both MD and M cohorts were then challenged with comparable dosages of the scabies mite *S. scabiei* var. *suis*, as described previously [28]. In brief, crusts, sourced from a single site of a heavily infested individual, were dissected into approximately 0.5 cm^2^ pieces containing a few hundred mites and inserted into the vertical ear canal of both ears of the piglets. The animals were temporarily restrained, which prevented dislodgement of the crusts by agitation and ensured successful infestation. Skin scrapings were taken every two weeks unless otherwise stated, from all cohorts to monitor the development of the infection from healthy to moderate and subsequently to severe status of disease. Scrapings were performed in the same manner for every pig, alternating ears and following a predetermined map to ensure maximal conformity and to avoid repeated sampling of the same site. In the case of severe infestation, crusts were first lifted and collected for mite isolation. Subsequently the exposed skin area was sampled. After sample collection at week 16, pigs in all cohorts including the mite-naive cohort C were treated with the acaricide Doramectin (1% ivermectin solution, Pfizer Animal Health) by intramuscular injection at the recommended dosage of 300 mg per kg of body weight. The skin was allowed to heal completely for 5 weeks after Doramectin treatment. Final skin scrapes were taken at week 21. All skin samples were collected in 2 ml reinforced centrifuge tubes (Precellys, Bertin Technologies) containing 200 µl of enzymatic lysis buffer (20 mM Tris, pH 8.0, 2 mM EDTA, 1.2% (v/v) Triton-X100) and stored at −80°C until further processing. Samples representing base line (week 0), mild infestation (week 7), severe infestation (week 10) and after healing (week 21) were taken at pigs' ages of 6, 13, 16, 27 weeks, respectively (Figure S1, supplemental information).

### Scabies mite collection from crusts

The skin crusts collected from severe scabies infections were placed in lidded glass petri dishes and incubated over a moderately warm light source, which allowed mites to leave the substrate. Between 50 and 200 mites were collected into 2 ml reinforced centrifuge tubes (Precellys, Bertin Technologies). Mites were washed twice for 7 minutes with shaking in 4% paraformaldehyde (PFD) followed by one wash in PBS to remove external bacteria. Mites were centrifuged for 2 min at 10,000 rpm, wash solutions were removed and the mite pellet was stored at −80°C.

### DNA extraction

The skin samples were first incubated in lysozyme (20 mg/ml) for 30 min in a 37°C water bath. Isolated mite samples were not treated with lysozyme. To facilitate homogenization, six 2.8 mm stainless steel beads (Precellys, Bertin Technologies) were added to both skin and mite samples. The samples were processed in a tissue homogenizer (Precellys24, Precellys, Bertin Technologies) at 6,800 rpm for 30 s. Beads were removed and DNA was extracted from the samples using the DNeasy Blood and Tissue Kit (Qiagen) according to the manufacturer's instructions with the minor modification as follows. Samples were first incubated with 200 µl of Buffer AL, 40 µl Proteinase K in a 56°C water bath overnight and the standard protocol was followed for all subsequent steps. Purified genomic DNA was eluted in 100 µl buffer AE and the purity and concentration of the samples were analyzed by a spectrophotometer, NanoDrop 2000 (Thermo Scientific). DNA samples were stored at −20°C until required.

### PCR amplification of 16S rDNA and data analysis

The bacterial 16S rDNA tag-encoded FLX-titanium amplicon pyrosequencing (bTEFAP) was contracted to Mr. DNA Molecular Research LP, Texas [31], [32]. Briefly, 16S rDNA was amplified from purified genomic DNA using the primer pair 27F (5' AGRGTTTGATCMTGGCTCAG 3')-519R (5' GTNTTACNGCGGCKGCTG 3') spanning V1-V3 region (∼500 bp product). The concentrations of all DNA samples were adjusted to a nominal 20 ng/µl and a 1 µl aliquot of each sample was used per 50 µl PCR reaction. A single-step fusion 30 cycle PCR using HotStarTaq Plus Master Mix Kit (Qiagen, Calencia, CA) was performed under the following conditions: 94°C for 3 min, followed by 28 cycles of 94°C for 30 s, 53°C for 40 s, 72°C for 1 min, and a final elongation step at 72°C for 5 min. Amplicon products from different samples were mixed in equal concentrations and purified using Agencourt Ampure beads (Agencourt Bioscience Corporation, MA, USA). Samples were then sequenced utilising Roche 454 FLX-titanium instruments and reagents following manufacturer's guidelines.

The 16S rDNA sequences were processed using the software packages QIIME 1.5[33]. Barcode sequences were removed by searching for exact matches and primer sequences were trimmed allowing 1 mismatch. Chimeras were removed using ChimeraSlayer [34]. Taxonomic assignments were retrieved by the RDP Classifer v2.2 [35] with a confidence threshold of 0.6. Operational taxonomic units (OTUs) were generated using the USEARCH package v5.2.32 [36] with an identity threshold of 97%. A representative sequence was selected for each OTU and taxonomically assigned with the RDP Classifier with a confidence cut-off of 0.6. Subsequently, statistical analysis was performed using R and the Calypso software (bioinfo.qimr.edu.au/calypso). The statistical analysis was done using one relative genus and OTU abundances, i.e. the number of reads assigned to each OTU or genus divided by the total number of reads obtained for each sample. Significant changes in the abundance of genera at different time points were detected by paired t-tests. Shannon index was used to estimate microbial community diversity (OTU level).

Representative sequences of OTUs assigned to *Staphylococcus* or *Streptococcus* by the RDP Classifier were used for phylogenetic analysis. Only OTUs with a relative abundance of at least 0.2% and 0.1% were included for the *Staphylococcus* and *Streptococcus* trees, respectively. Multiple alignments of reference 16S sequences of the corresponding genera were retrieved from the RDP database [37] and used as a reference to align the representative OTU sequences using HMMER3 [38], [39]. Phylogenetic trees were reconstructed using FastTree [40] with a generalized time-reversible (GTR) model.

## Results and Discussion

### Trial summary

All control animals (C group) remained clear of mites and healthy throughout the entire trial. At week 7 all scabies treated animals (M and MD groups) showed symptoms of successful mite infestation, such as a typical rash over large parts of the body leading to scratching behavior. No other symptoms of compromised health were detected in the M and MD animals. Severe infestations with crust formation in the inner part of the ear pinnae were seen between weeks 10 and 16, allowing isolation of mites. Crusts were formed in one member of the M group and in all animals treated with Dexamethasone (MD group). The antiparasitic drug Doramectin^®^ was administered by intramuscular injection at the start of week 16, at a recommended dosage to kill off the mites within a week [41]. Infected skin healed within 5 weeks before the final scrapings were taken at week 21. A total of 140 skin samples were collected over the 21 week trial period. The sampling site was restricted to the inner sebaceous surface of the ear pinnae because this is the primary site of early scabies infestations in pigs. Ninety six samples were subjected for pyrosequencing bacterial 16S rDNA, yielding 1,366,477 high quality 16S sequences with an average length of 407 base pairs. Sequence data has been deposited at http://www.ncbi.nlm.nih.gov/sra with the accession number SRX392076. Samples taken from week 2 were excluded due to redundancy. A remaining subset of 57 samples collected in weeks 0, 7, 10, 13, 16 and 21 yielding 744,225 16S sequences were subjected to further analysis.

### The healthy skin microbiota in pre-adult pigs

**Figure 1:**
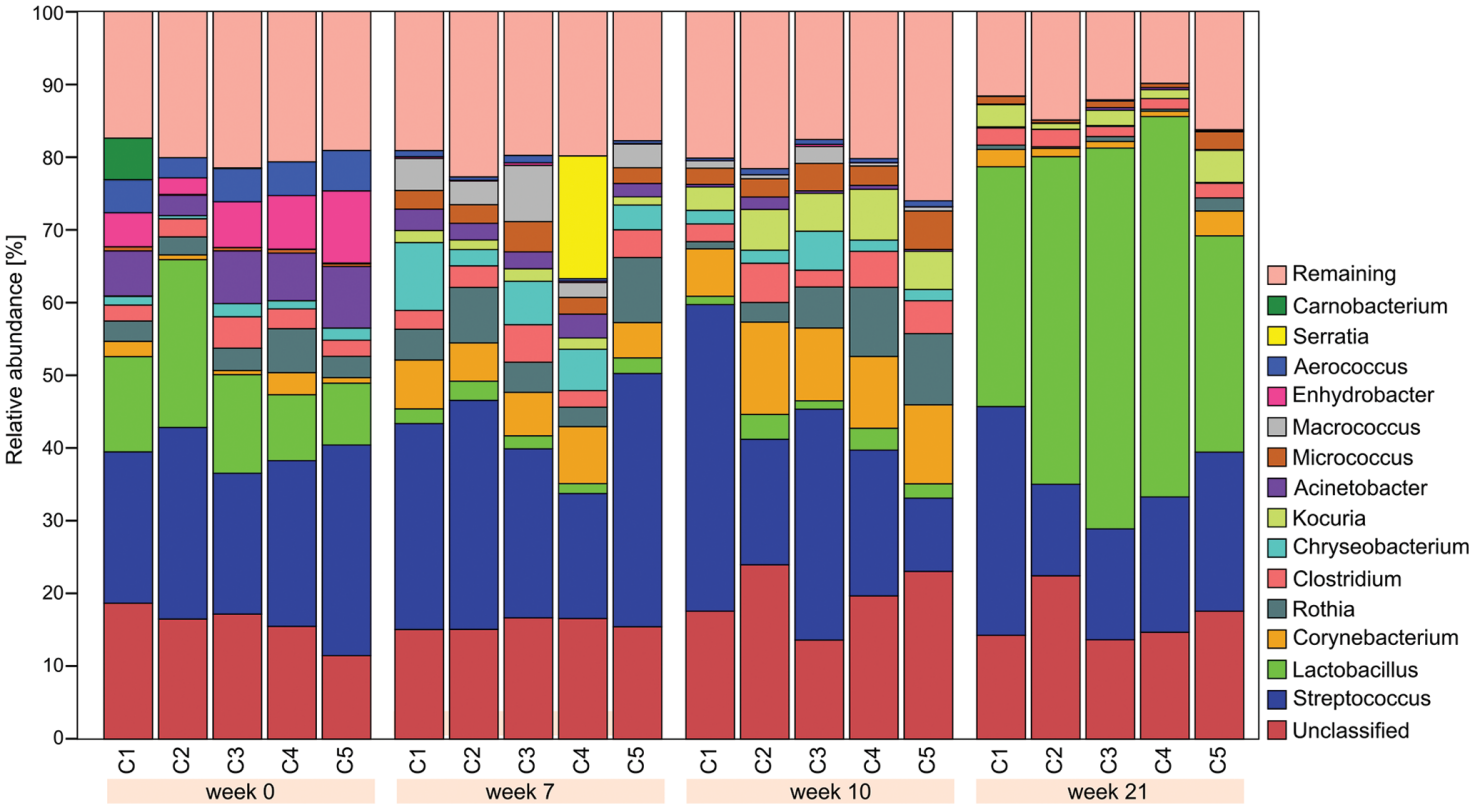
Relative abundance of the most predominant bacterial genera in the pinna of porcine ears at various time points in the absence of mites. At week 0, *Streptococcus* was the most dominant genus, followed by *Lactobacillus, Actinectobacter, Enhydrobacter, Aerococcus* and *Rothia* in decreasing abundance. At week 7, *Streptococcus* was still the most dominant genus, followed by smaller abundance of *Corynebacterium, Chryseobacterium, Rothia, Clostridium* and *Lactobacillus*. Similarly at week 10, *Streptococcus* was the dominant genus, followed by *Corynebacterium, Rothia, Kocouria, Clostridium* and *Lactobacillus*. At week 21, *Lactobacillus* was the most dominant, followed by *Streptococcus* and *Kocuria*. Only genera, with a relative abundance of ≥10% in at least one sample are illustrated.

**Figure 2:**
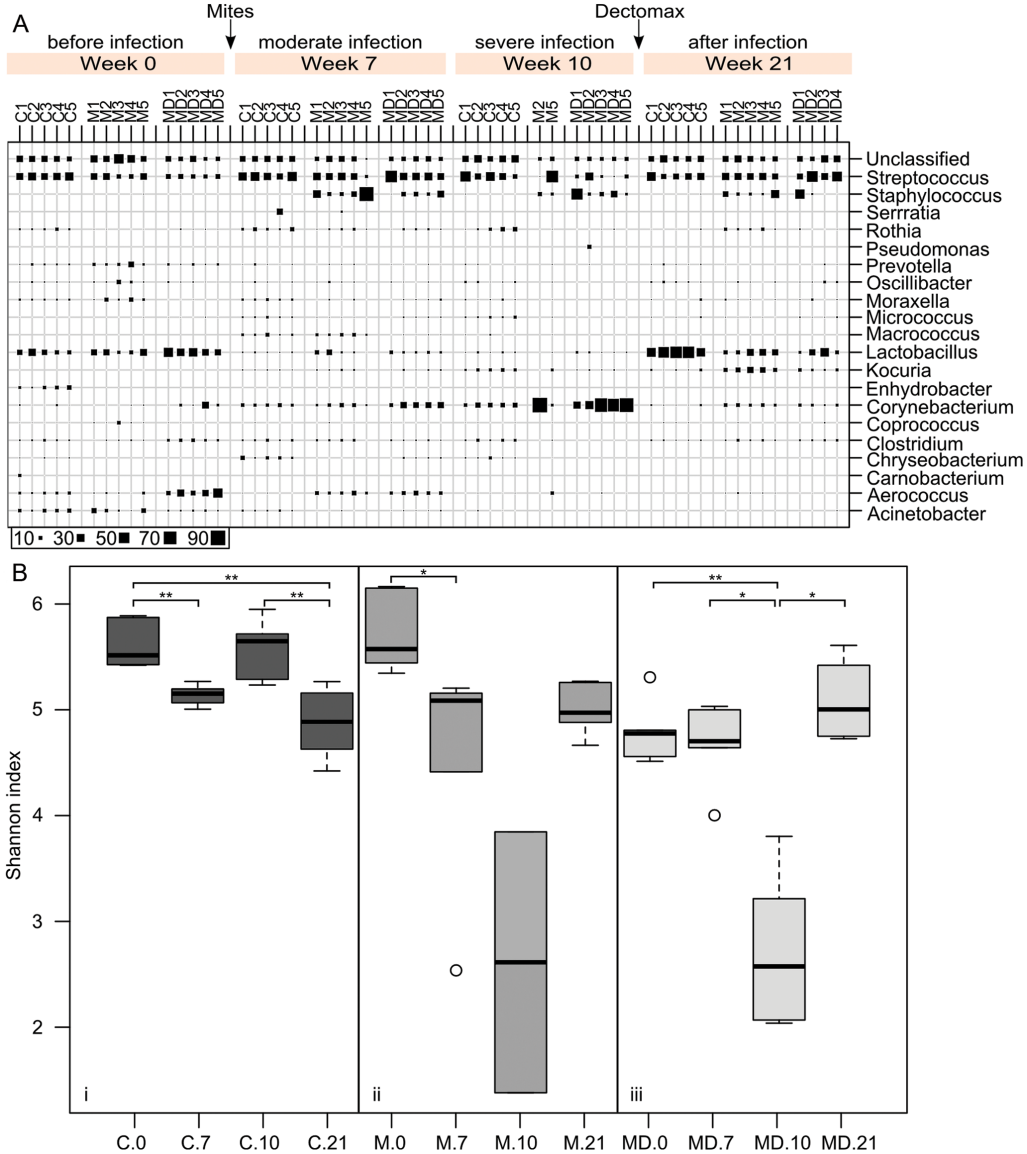
(a) Composition of the microbial communities before, during and after scabies mite infection. Microbial community structure was analysed by 454 pyrosequencing of the 16S rRNA gene of the total DNA, extracted from the skin scrapings of the pinna of pig ears. Samples were taken before the scabies infection from all cohorts at week 0. Cohorts M and MD were then challenged with an equal dosage of *S. scabiei* var. *suis* in both ears. Visible signs of scabies infection developed by week 7, which progressed to severe form of infection by week 10. Scabies infection was treated with an acaricide (Dectomax^®^) and skin was allowed to heal. Final skin scrapings were taken at week 21. Bubble volumes correlate with the abundance (%) of the each genus detected in the sample. (**b**) Effect of scabies mite infection on the microbial community diversity visualised by Shannon index for all samples from the cohorts C (i, mite free), M (ii, mite infested), MD (iii, mite infested and Dexamethasone treated). Community diversity is expressed as the mean Shannon index (OTU level). Paired t-test was carried out for pigs where both time points were available (M.10: n = 2, MD: n = 4, other: n = 5). Significant differences are annotated by *: p<0.05, **:p<0.01, ***: p<0.001.

Twenty samples from the inner sebaceous surface of the ear pinnae were obtained from the Control cohort C containing on average 8,053 sequences per sample (range: 5,036–30,698). In these twenty skin samples we identified 204 different bacterial genera with at least 5 assigned sequences. The major genera are listed in Table 1. We observed significant changes in the skin microbiota of healthy animals during the 21 weeks period of the trial (Figure 1). At week 0, *Streptococcus* was the most abundant genus of the skin microbiota of healthy pigs (23% of 16S sequences) followed by *Lactobacillus* (13%) (Table 1, Figure 1). While *Streptococcus* remained relatively constant throughout the trial, *Lactobacillus* transiently dropped in abundance to 2% in weeks 7 and 10 (p = 0.01), but then became the most abundant genus with 45% of 16S sequences at week 21 (p = 0.001). We also observed a considerable reduction in microbial diversity: *Lactobacillus* and *Streptococcus* together represented about 30% of sequences at week 0 and had risen to about 70% at week 21, thereby having largely replaced the next 10 abundant genera. This change in community composition was reflected in a reduction of the community diversity measured by Shannon index. The Shannon index at week 21 was significantly lower than in weeks 0 (p = 0.009) and 10 (p = 0.008) (Figure 2bi) and the evenness had dropped from 0.75 to 0.66 (Figure S2, supplemental information). During the first 10 weeks the microbial diversity fluctuated only moderately, as indicated by similar Shannon indices (Figure 2bi).

**Table 1:** Relative abundance of the bacterial genera at defined time points.

Genus	C0	C7	C10	C21	M0	M7	M10	M21	MD0	MD7	MD10	MD21
*Acinetobacter*	6.5	2.3	0.4	0.1	6	0.55	0	0.1	2.6	1	0.1	0.4
*Aerococcus*	4.6	0.4	0.7	0	1.5	6.4	0.9	1.15	17.8	6.8	0.6	0.45
*Carnobacterium*	1.16	0	0	0	0	0	0	0	0	0	0	0
*Chryseobacterium*	1.24	5.32	2.4	0.08	0.2	0.12	0.175	1.76	0.1	0.167	0.033	0.4
*Clostridium*	2.5	3	4.5	2	1.6	3.05	0.3	3	4.6	1.9	0.2	2.9
*Coprococcus*	0.7	0.5	0.7	0.9	1.5	0.55	0	0.45	0.6	0.5	0	0.3
*Corynebacterium*	0.8	6	10	1.1	0.6	7	73.5	6	4.2	15.2	62.7	4.6
*Enhydrobacter*	6.3	0.2	0	0	0	0.1	0	0	0	0.3	0	0
*Kocuria*	0	1.6	5.3	2.1	0.2	2.3	0.3	11.95	0.1	2.3	1.3	4.45
*Lactobacillus*	13.1	2	2	45	16.1	2.9	0.1	14.5	24.1	3.8	1.8	12.1
*Macrococcus*	0	3.3	0.6	0.1	0	5.65	0.1	0.25	0	1	0.1	0.15
*Micrococcus*	0.5	2.5	2.7	0.9	0.2	0.75	0.1	0.75	0.6	1.6	0.5	1.4
*Moraxella*	2.7	3.4	1.4	0.4	3.9	1.35	0.1	1.05	0.4	1.5	0.1	1.2
*Oscillibacter*	1.7	1.3	2.7	2.4	2	1.5	0	1.4	1.3	1	0.1	2.25
*Prevotella*	2.6	1.1	0.6	1.3	5.3	0.9	0	0.5	3.3	0.6	0.1	0.7
*Pseudomonas*	0.08	0.02	0.02	0.02	0.06	1.78	0	0	0.02	0	0.017	0.067
*Rothia*	2.9	4.2	5.6	0.6	0.7	2.95	0.2	3.25	0.9	3.1	0.4	0.6
*Serratia*	0	3.3	0	0	0	0	0	0	0	0.5	0	0
*Staphylococcus*	0.3	0.1	0.4	0.5	0.5	11	8.9	5.5	0.3	9.8	9.4	1.7
*Streptococcus*	22.8	28.3	20	18.6	18.8	19.7	1.5	21.2	10.9	23.8	7.2	29.35
Unclassified	16.5	15.4	19.7	14.7	22	13.7	7.6	15.65	10.2	10.3	8.9	17.25

Numbers are presented as the median percentage of the relative abundances measured for five samples in each cohort M (mite infected), MD (Dexamethasone treated and mite infected) and C (mite-free control).

In summary, the skin microbiota became gradually less diverse as the healthy piglets matured, reducing from a complex and diverse assemblage during the juvenile stage to two dominating genera, i.e. *Lactobacillus* and *Streptococcus* at week 21. While there is no comparable data from human infants, Grice *et al*. have previously reported that bacterial communities in adult human sebaceous microenvironments were less diverse than in other skin sites [42].

### The effect of *S. scabiei* var. *suis* infection on the skin microbiota in pre-adult pigs

In total, 57 samples with a median of 8,798 sequences (range: 2,467–55990) were included to study changes of the skin microbiota associated with scabies. Prior to *S. scabies* infection in week 0 the control (C) and scabies treated (M) cohorts showed similar skin microbial community profiles, whereas the skin microbiota of the scabies+Dexamethasone treated cohort (MD) was significantly different. *Streptococcus* and *Lactobacillus* were the most dominant genera in cohorts C and M (Figure 2a) but *Lactobacillus* (24.9%) and *Aerococcus* were the most abundant genera within the cohort MD. Also, the microbial diversity was significantly lower in the MD cohort compared to the M and C cohorts (Figure 2b). Dexamethasone treatment has previously been reported to improve neutrophil-mediated killing of streptococci in a rat animal model [43], which could explain the observed differences in community composition. Notably, Dexamethasone treatment did not impact on the main changes in the skin microbiota seen in mite infested animals, as outlined below.

At week 0, before scabies mites were introduced, only a small percentage (≤0.5%) of *Staphylococcus* was present in all three cohorts (Figure 2a). During moderate scabies infection at week 7, a major increase in *Staphylococcus* was observed. At week 7, *Staphylococcus* abundance ranged from 6% to 76% in cohort M, and from 1% to 20% in cohort MD (Figure 2a). In contrast, *Staphylococcus* was nearly absent in the mite-free control cohort C, ranging from 0% to 0.2%. During severe scabies infestation (week 10), similar abundance of *Staphylococcus* remained in M and MD cohorts as in week 7, with one animal of the MD cohort reaching to 49%, compared with low numbers (0.2% to 1%) in the mite-free control cohort C (Figure 2a).

In week 10, when crust formation had occurred in one individual of the M cohort (M2) and all animals of the MD cohort, a dramatic reduction in community diversity was observed in samples taken from crusted sites in these animals (Figures 2bii and 2biii). The microbial community profiles from these skin samples, which were highly infested with mites, comprised up to 80% of *Corynebacterium* at the expense of other genera (Figure 2a).

In week 16, pigs were treated with the antiparasitic drug Doramectin. At week 21, after the mite infection was cleared in cohorts M and MD, the skin microbiota showed a similar diversity in all three cohorts (Figure 2b). However, the relative abundance of individual genera was markedly different between the control cohort C and the previously mite infected groups M and MD (Figure 2a). *Staphylococcus* remained abundant in most samples of the previously mite infected animals (reaching 27.4% in cohort M and 35.5% in cohort MD) compared to a minuscule presence of 0.2 to 1% in the control animals (Figure 2a). At the same time *Lactobacillus* was present at low levels in cohorts M (14%, Table 1) and MD (12%) while it was the dominant genus in the control cohort C (45%). *Streptococcus* was more dominant in the previously mite infected groups M (21%) and MD (29%) compared to the control cohort C (18.6%). At week 21, when all pigs were free of mites, *Corynebacterium* was equal to or below 6% in all cohorts.

### Alteration in the staphylococcal population during scabies mite infection

**Figure 3:**
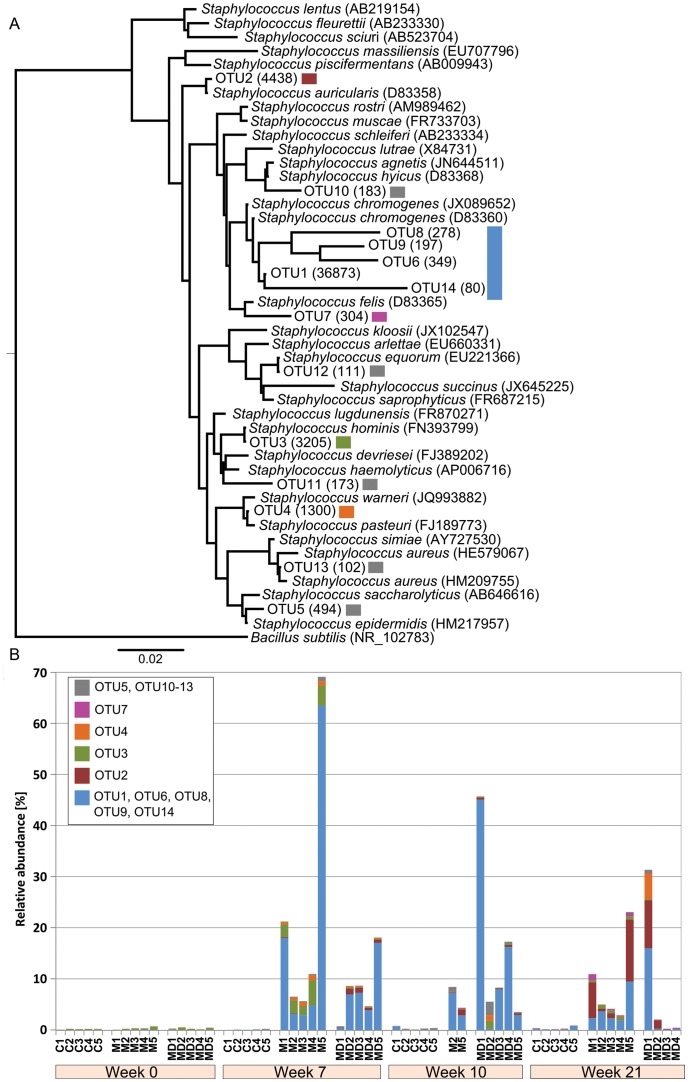
(a) Phylogenetic tree of *Staphylococcus* indicating tentative species. **(b)** Relative abundance of staphylococcal species in each sample. (**a**) A phylogenetic tree was constructed for OTUs assigned to the genus *Staphylococcus* using known reference 16S rRNA genes. The accession is noted in bracket for each reference species. The number in brackets following the OTU name indicates the read counts within the OTU. The tree was rooted with the outlier *Bacillus subtilis*. Several OTUs (1, 6, 8, 9, 14) are in close proximity to the 16S rRNA gene of *S. chromogenes*. (**b**) Bar chart demonstrating the relative abundance of each OTU in the pig cohorts over time. An extensive change of the OTUs is measured in the scabies infected cohort M and MD from week 7 onwards. The change was irreversible despite treatment. In particular, OTU1, OTU6, OTU8, OTU9, OTU14, all related to *S. chromogenes*, are dominating the skin microbiota.

The significant apparent increase in the *Staphylococcus* abundance in the scabies infected pigs indicated a correlation between *S. scabiei* infestations and *Staphylococcus* growth possibly combined with selective removal of other genera. To further identify the tentative species of *Staphylococcus* present, we constructed a phylogenetic tree of pig skin OTUs and known *Staphylococcus* species (Figure 3a). Prior to trial commencement at week 0, an OTU closely related to *S. hominis* (OTU3) was present at a very low abundance across all cohorts (Figure 3b). *Staphylococci* remained low throughout the trial in the scabies free control C. In contrast, from week 7 onwards *Staphylococcus* abundance significantly increased in cohorts M and MD and the community shifted from OTU3 to OTUs 1, 6, 8, 9 and 14, which are closely related to *S. chromogenes* (Figures 3a and 3b). After the scabies mites had been killed by drug treatment at week 16, staphylococci persisted in week 21 in cohorts M and MD. Two major taxonomic units, OTU1 and OTU2 (closely related to *S. chromogenes and S. auricularis* respectively) were in high abundance, followed by a lower abundance in OTU3, OTU4 (closely related to *S. hominis* and *S. pasteuri*) and OTU7 (closely related to *S. felis*) (Figure 3b).

Staphylococci are part of the healthy skin microflora but are also common causative agents of pyoderma in pigs and other animals [44]–[46], including humans [47], with different species predominating in pig and human skin [42], [48], [49]. *S. hominis* is a normal skin commensal of human and animal skin, whereas *S. chromogenes* is recognized as the causative agent of exudative epidermitis in pigs [45]. Further, *S. chromogenes* was described to play a role in skin lesions, dermatitis and otitis media in sheep, associated with infestation by the sheep scab mite *Psoroptes ovis*, and has been identified in the skin microbiota associated with *P. ovis* [50]. *S. auricularis*, *S. pasteruri* and *S. felis* are coagulase negative, skin residents of human and animals, and were reported to occasionally cause diseases [39], [51]–[53]. *S. epidermidis* prevails on healthy human skin while *S. aureus* is considered an important primary pathogen. In contrast, *S. aureus* was identified as the predominant species on healthy adult pig skin while *S. epidermidis* was less frequent [48]. A global increase in transmission of pathogenic methicillin resistant *S. aureus* strains has been reported between humans and animals [54]. In humans the link between scabies and *S. aureus* infections is well documented, however exclusively in epidemiological studies [55]–[60]. On human skin *S. epidermidis* usually has a benign relationship with its host [61] and was proposed to have a protective role in preventing colonisation with pathogenic bacteria, such as *S. aureus* [62]. Since *S. aureus* and *S. epidermidis* had been isolated from pigs before [48] we expected to detect these species on healthy skin and/or an increase in *S. aureus* during scabies infection; however neither was the case. Their absence in this experimental setting may be due to the sampling site being the sebaceous pinnae of the ears and not the back of the pigs, where they were detected previously [48]. Moreover, *S. aureus* likely becomes more abundant on the porcine skin after environmental exposure and human contact when housed under normal farming conditions [48]. The piglets in this trial were housed in a controlled facility without contact to other animals or humans, except for handlers who wore gloves and freshly washed overalls for every procedure.

Notably, in the trial presented here, staphylococci were abundant only in the pigs that had encountered scabies mites (cohorts M and MD), but barely measurable in the microbiome of mite free pigs in the control cohort C (Figure 3b). The presence of *Staphylococcus* in large abundance unveils an obvious risk factor for future recurrent bacterial infections in scabies infected animals. While the overall microbial diversity of the mite free skin in control cohort C was similar to that of scabies infected skin in cohorts M and MD, the significant increase of the genus *Staphylococcus* in the skin of scabies infected pigs strongly suggests that the mite infection selectively favored the establishment of staphylococci. Complement appears to be a major primary defense mechanism of the vertebrate host targeted at mites as well as bacteria. Scabies mites release proteins that inhibit complement [16], [17] and by reducing complement defense in their vicinity the mites may provide a microenvironment that fosters the survival of pathogenic bacteria [18]. *S. aureus* also displays an impressive arsenal of complement interference mechanisms [63]. Intriguingly, during human scabies infestations a substantial increase of *S. aureus* pyoderma is observed [64], implying that the combined presence of mites and bacteria may further amplify the inflammation response. Similarly, the dominance of the pathogenic *S. chromogenes* over the commensal *S. hominis* in the mite infested pig skin may be driven by the production of a range of virulence factors produced by *S. chromogenes* and the mites.

### Detailed analysis of the resident *Streptococcus*

**Figure 4:**
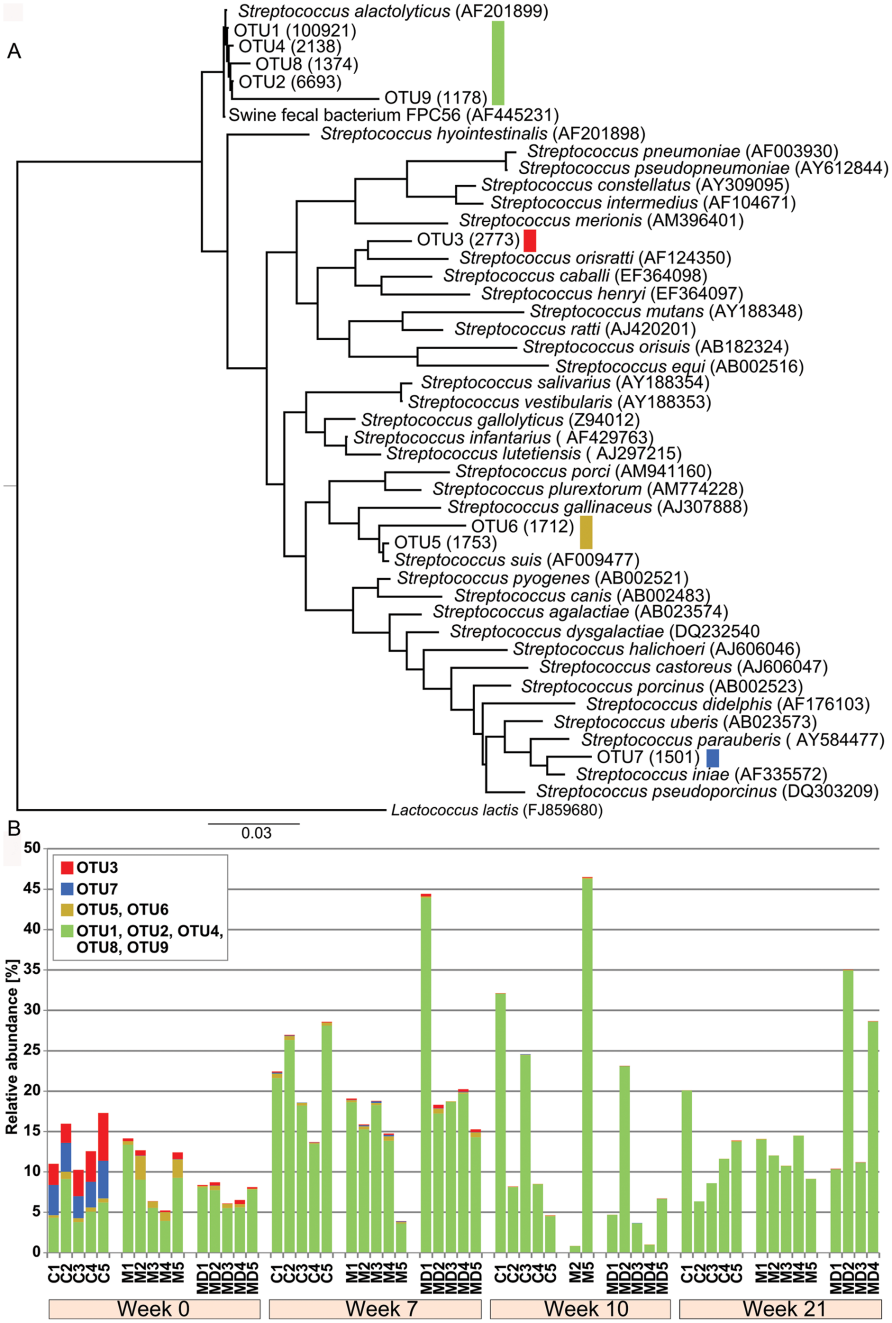
(a) Phylogenetic tree of *Streptococcus* indicating tentative species. **(b)** Relative abundance of streptococcal species in each sample. (**a**) A phylogenetic tree was constructed for OTUs assigned to the genus *Streptococcus* using known reference 16S rRNA genes. The accession is noted in bracket for each reference species. The number in brackets following the OTU name indicates the read counts within the OTU. The tree was rooted with the outlier *Lactococcus lactis*. (**b**) Bar chart demonstrating the relative abundance of each OTU in the pig cohorts over time. The graph depicts that the OTUs are overall stable between the three cohorts within each week and over time, irrespective of the presence or absence of mites.

*S. pyogenes* is another important human pathogen commonly isolated from scabies associated pyoderma in human patients, a species that has not been reported in pigs. However, the genus *Streptococcus* was the most stable genus and relatively prominent in almost all skin samples throughout the duration of this trial (Figure 2a). Importantly, this *Streptococcus* population was not severely affected by *S. scabiei* infection, presenting at a constant abundance prior to, during and after the scabies mite infection. A phylogenetic tree of *Streptococcus* was constructed to study changes on OTU level (Figure 4a). OTUs closely related to *S. alactolyticus* dominated the streptococcal population (OTUs 1, 2, 4, 8 and 9) (Figure 4b). *S. alactolyticus* is considered to be part of the normal gut microbiota of pigs and has been isolated from gastrointestinal tracts of newly weaned piglets and feces [65], [66]. Our data showed that *S. alactolyticus* is also likely part of normal skin microbiota in juvenile pig ears. Interestingly OTU5 and 6 most similar to an opportunistic pathogen *Streptococcus suis* was present in a small proportion in the majority of the samples analyzed, but did not increase at any time point. *S*. *suis* is primarily an opportunistic pathogen of pigs but also an emerging human pathogen in the tropics [67]. Although *S. suis* is known to cause diseases in humans, particularly among pig handlers [68], our results suggest that handling scabatic pigs may not pose additional risk in this regard.

### *Corynebacterium* abundance is associated with mite bodies

**Figure 5:**
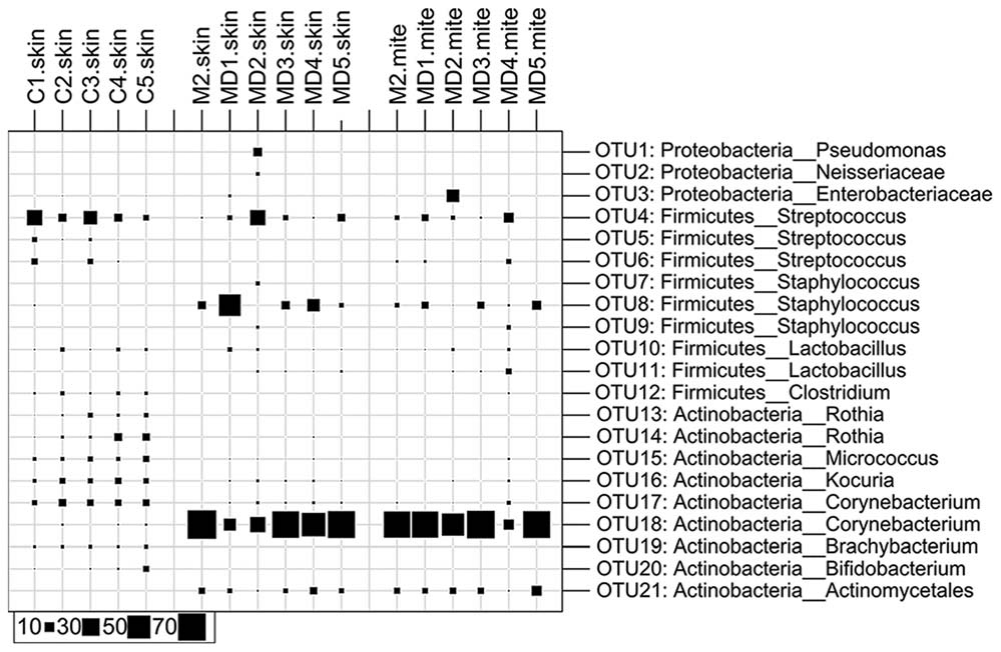
Microbial composition of skin samples and isolated scabies mites collected from severely scabies infested crusted sites (week 10) and control animals. Bubble plot of the taxonomic profile as calculated for the OTU level. Only OTUs with a relative abundance of ≥2% in at least one sample are shown. Each OTU is denoted with the taxonomic classification according to the RDP Classifier (confidence value ≥0.6). The taxonomic composition between the healthy skin and the mite containing skin as well as the washed mites is differing mainly based on the relative abundance of *Corynebacteria*.

In week 10, when crust formation had occurred in individuals M2 and MD1–5, a dramatic reduction in community diversity was observed (Figures 2bii and 2biii). Skin samples taken from crusted sites comprised up to >70% of *Corynebacterium* at the expense of other genera (Figure 5). While in an ordinary scabies skin scraping sample even a single mite is very rarely seen, the immediate skin layer below a crust generally contains high numbers of mites, which together with their internal bacteria are part of the skin microbiota. The samples taken from crusted areas in week 10 were heavily infested with mites. Coincidently, a high proportion of the same *Corynebacterium* sequences were also seen in samples generated from isolated washed mites, indicating that *Corynebacteria* are part of the mite internal microbiota. Consequently, the high abundance of *Corynebacterium* sequences detected in the skin scrapings taken from the severely infected sites is likely due to the mite internal microbiota from mites present in the scraping. At week 21 the previously mite infected but now mite free cohorts M and MD showed low levels of *Corynebacterium* (Figure 2a, Table 1), reinforcing the hypothesis that this genus could be enriched predominantly within the gut of the mites themselves. Members of this genus are facultative anaerobes and hence well suited to the locally lowered O_2_ within the mite gut beneath dense skin crusts. Symbiotic *Corynebacteria* have been isolated from the alimentary systems of a variety of arthropods feeding on skin, such as *Triatoma infestans* [69] and the tick species *Ixodes ricinus*, *Dermacentor reticulatus* and *Haemaphysalis concinna* [52], providing potential novel strategies to control the transmission of diseases [70]. Thus, a subsequent study will focus on a thorough characterization of the scabies mite-internal microbiome and potential symbionts which could be targeted for scabies control.

### Summary and conclusions

We demonstrated that scabies infestation has an impact on the host's skin microbiota. In an experimental porcine skin model, abundance of potentially pathogenic *Staphylococcus* species increased with the onset of infection, and remained beyond treatment and healing. At the end of the trial previously scabies infested animals showed a much reduced presence of *Lactobacillus* compared to the control animals. The local *Streptococcus* population remained stable in all cohorts throughout the trial and seemed almost unaffected by scabies. *Corynebacterium* abundance in heavily mite infested skin samples was possibly related to the mite-internal microflora.

This study provides the first *in vivo* demonstration of a mite induced shift in the healthy skin microbiota, supporting direct evidence of the previously alleged link between scabies and pyoderma due to *Staphylococcu*s infections, as seen in humans [9], [11]–[13]. The study focused on the primary site of mite infestations in young piglets and the experimental setup allowed monitoring of the site over time, i.e. prior to, during and after scabies mite infestation. It provides a basis for future investigation in human patients. The study highlights that scabies mite infestation is not a simple ‘itch’ but should be viewed as a complex disease involving a change in the status of the skin microbiota, which gives rise to serious secondary infections.

## Supporting Information

Figure S1 **Timeline of experimental trial to monitor the scabies associated microbiota in the porcine model correlating relevant trial time points with the pigs' ages.**
(TIF)

Figure S2 **Effect of scabies mite infection on the evenness index for all samples from the cohorts C (i, mite free), M (ii, mite infested), MD (iii, mite infested and Dexamethasone treated). **Evenness index is expressed as the Pielou's evenness (OTU level). Paired t-test was carried out for pigs where both time points were available (M.10: n = 2, MD: n = 4, other: n = 5). Significant differences are annotated by *: p<0.05, **:p<0.01, ***: p<0.001.
(TIF)

## References

[1] Engelman D, Kiang K, Chosidow O, McCarthy J, Fuller C, et al (2013) Toward the Global Control of Human Scabies: Introducing the International Alliance for the Control of Scabies. PLoS Negl Trop Dis 7(8): e2167 doi:10.1371/journal.pntd.0002167 23951369 10.1371/journal.pntd.0002167 PMC3738445

[2] Hay RJ, Steer AC, Engelman D, Walton S (2012) Scabies in the developing world—its prevalence, complications, and management. Clin Microbiol Infect 18: 313–323. 22429456 10.1111/j.1469-0691.2012.03798.x

[3] Fuller LC (2013) Epidemiology of scabies. Curr Opin Infect Dis 26: 123–126. 23411418 10.1097/QCO.0b013e32835eb851

[4] Hicks MI, Elston DM (2009) Scabies. Dermatol Ther 22: 279–292. 19580575 10.1111/j.1529-8019.2009.01243.x

[5] Makigami K, Ohtaki N, Ishii N, Yasumura S (2009) Risk factors of scabies in psychiatric and long-term care hospitals: a nationwide mail-in survey in Japan. J Dermatol 36: 491–498. 19712276 10.1111/j.1346-8138.2009.00691.x

[6] Lassa S, Campbell MJ, Bennett CE (2011) Epidemiology of scabies prevalence in the U.K. from general practice records. Br J Dermatol 164: 1329–1334. 21574970 10.1111/j.1365-2133.2011.10264.x

[7] Mahe A, Hay RJ (2005) Epidemiology and management of common skin diseases in childern in developing countries. World Health Organization Discussion papers on child health.

[8] Whitehall J, Kuzulugil D, Sheldrick K, Wood A (2013) Burden of paediatric pyoderma and scabies in North West Queensland. J Paediatr Child Health 49: 141–143. 23347222 10.1111/jpc.12095

[9] Clucas DB, Carville KS, Connors C, Currie BJ, Carapetis JR, et al (2008) Disease burden and health-care clinic attendances for young children in remote aboriginal communities of northern Australia. Bull World Health Organ 86: 275–281. 18438516 10.2471/BLT.07.043034 PMC2647416

[10] Hay RJ, Steer AC, Chosidow O, Currie BJ (2013) Scabies: a suitable case for a global control initiative. Curr Opin Infect Dis 26: 107–109. 23302759 10.1097/QCO.0b013e32835e085b

[11] Hengge UR, Currie BJ, Jager G, Lupi O, Schwartz RA (2006) Scabies: a ubiquitous neglected skin disease. Lancet Infect Dis 6: 769–779. 17123897 10.1016/S1473-3099(06)70654-5

[12] Andrews RM, Kearns T, Connors C, Parker C, Carville K, et al (2009) A regional initiative to reduce skin infections amongst aboriginal children living in remote communities of the Northern Territory, Australia. PLoS Negl Trop Dis 3: e554. 19936297 10.1371/journal.pntd.0000554 PMC2775159

[13] Steer AC, Jenney AW, Kado J, Batzloff MR, La Vincente S, et al (2009) High burden of impetigo and scabies in a tropical country. PLoS Negl Trop Dis 3: e467. 19547749 10.1371/journal.pntd.0000467 PMC2694270

[14] Carapetis J, Steer AC, Mulholland EK (2005) The current evidence for the burden of group A streptococcal diseases. World Health Organization Discussion papers of child health.

[15] McCarthy JS, Kemp DJ, Walton SF, Currie BJ (2004) Scabies: more than just an irritation. Postgrad Med J 80: 382–387. 15254301 10.1136/pgmj.2003.014563 PMC1743057

[16] Bergstrom FC, Reynolds S, Johnstone M, Pike RN, Buckle AM, et al (2009) Scabies mite inactivated serine protease paralogs inhibit the human complement system. J Immunol 182: 7809–7817. 19494305 10.4049/jimmunol.0804205

[17] Mika A, Reynolds SL, Mohlin FC, Willis C, Swe PM, et al (2012) Novel scabies mite serpins inhibit the three pathways of the human complement system. PLoS One 7: e40489. 22792350 10.1371/journal.pone.0040489 PMC3394726

[18] Mika A, Reynolds SL, Pickering D, McMillan D, Sriprakash KS, et al (2012) Complement inhibitors from scabies mites promote streptococcal growth—a novel mechanism in infected epidermis? PLoS Negl Trop Dis 6: e1563. 22815998 10.1371/journal.pntd.0001563 PMC3398963

[19] Kong HH (2011) Skin microbiome: genomics-based insights into the diversity and role of skin microbes. Trends Mol Med 17: 320–328. 21376666 10.1016/j.molmed.2011.01.013 PMC3115422

[20] Morsy GH, Turek JJ, Gaafar SM (1989) Scanning electron microscopy of sarcoptic mange lesions in swine. Vet Parasitol 31: 281–288. 2527435 10.1016/0304-4017(89)90078-2

[21] Steinstraesser L, Vranckx JJ, Mohammadi-Tabrisi A, Jacobsen F, Mittler D, et al (2006) A novel titanium wound chamber for the study of wound infections in pigs. Comp Med 56: 279–285. 16941955

[22] Sullivan TP, Eaglstein WH, Davis SC, Mertz P (2001) The pig as a model for human wound healing. Wound Repair Regen 9: 66–76. 11350644 10.1046/j.1524-475x.2001.00066.x

[23] Hollander DA, Erli HJ, Theisen A, Falk S, Kreck T, et al (2003) Standardized qualitative evaluation of scar tissue properties in an animal wound healing model. Wound Repair Regen 11: 150–157. 12631304 10.1046/j.1524-475x.2003.11212.x

[24] Vodicka P, Smetana K Jr, Dvorankova B, Emerick T, Xu YZ, et al (2005) The miniature pig as an animal model in biomedical research. Ann N Y Acad Sci 1049: 161–171. 15965115 10.1196/annals.1334.015

[25] Salvesen B, Mollnes TE (2009) Pathway-specific complement activity in pigs evaluated with a human functional complement assay. Mol Immunol 46: 1620–1625. 19328551 10.1016/j.molimm.2009.02.028

[26] Jiang H, Zhang HM, Frank MM (2010) Subcutaneous infusion of human C1 inhibitor in swine. Clin Immunol 136: 323–328. 20627701 10.1016/j.clim.2010.05.001

[27] Meurens F, Summerfield A, Nauwynck H, Saif L, Gerdts V (2012) The pig: a model for human infectious diseases. Trends Microbiol 20: 50–57. 22153753 10.1016/j.tim.2011.11.002 PMC7173122

[28] Mounsey K, Ho MF, Kelly A, Willis C, Pasay C, et al (2010) A tractable experimental model for study of human and animal scabies. PLoS Negl Trop Dis 4: e756. 20668508 10.1371/journal.pntd.0000756 PMC2907415

[29] Lo DY, Lee WM, Chien MS, Lin CC, Lee WC (2005) Effects of dexamethasone on peripheral blood mononuclear cell phenotype in weanling piglets. Comp Immunol Microbiol Infect Dis 28: 251–258. 15896844 10.1016/j.cimid.2005.03.001

[30] Marliere V, Roul S, Labreze C, Taieb A (1999) Crusted (Norwegian) scabies induced by use of topical corticosteroids and treated successfully with ivermectin. J Pediatr 135: 122–124. 10393619 10.1016/s0022-3476(99)70342-2

[31] Dowd SE, Callaway TR, Wolcott RD, Sun Y, McKeehan T, et al (2008) Evaluation of the bacterial diversity in the feces of cattle using 16S rDNA bacterial tag-encoded FLX amplicon pyrosequencing (bTEFAP). BMC Microbiol 8: 125. 18652685 10.1186/1471-2180-8-125 PMC2515157

[32] Hooda S, Vester Boler BM, Kerr KR, Dowd SE, Swanson KS (2013) The gut microbiome of kittens is affected by dietary protein:carbohydrate ratio and associated with blood metabolite and hormone concentrations. Br J Nutr 109: 1637–1646. 22935193 10.1017/S0007114512003479

[33] Caporaso JG, Kuczynski J, Stombaugh J, Bittinger K, Bushman FD, et al (2010) QIIME allows analysis of high-throughput community sequencing data. Nat Methods 7: 335–336. 20383131 10.1038/nmeth.f.303 PMC3156573

[34] Haas BJ, Gevers D, Earl AM, Feldgarden M, Ward DV, et al (2011) Chimeric 16S rRNA sequence formation and detection in Sanger and 454-pyrosequenced PCR amplicons. Genome Res 21: 494–504. 21212162 10.1101/gr.112730.110 PMC3044863

[35] Wang Q, Garrity GM, Tiedje JM, Cole JR (2007) Naive Bayesian classifier for rapid assignment of rRNA sequences into the new bacterial taxonomy. Appl Environ Microbiol 73: 5261–5267. 17586664 10.1128/AEM.00062-07 PMC1950982

[36] Edgar RC (2010) Search and clustering orders of magnitude faster than BLAST. Bioinformatics 26: 2460–2461. 20709691 10.1093/bioinformatics/btq461

[37] Cole JR, Wang Q, Cardenas E, Fish J, Chai B, et al (2009) The Ribosomal Database Project: improved alignments and new tools for rRNA analysis. Nucleic Acids Res 37: D141–145. 19004872 10.1093/nar/gkn879 PMC2686447

[38] Eddy SR (1998) Profile hidden Markov models. Bioinformatics 14: 755–763. 9918945 10.1093/bioinformatics/14.9.755

[39] Eddy SR (2008) A probabilistic model of local sequence alignment that simplifies statistical significance estimation. PLoS Comput Biol 4: e1000069. 18516236 10.1371/journal.pcbi.1000069 PMC2396288

[40] Price MN, Dehal PS, Arkin AP (2009) FastTree: computing large minimum evolution trees with profiles instead of a distance matrix. Mol Biol Evol 26: 1641–1650. 19377059 10.1093/molbev/msp077 PMC2693737

[41] Yazwinski TA, Tucker C, Featherston H, Johnson Z, Wood-Huels N (1997) Endectocidal efficacies of doramectin in naturally parasitized pigs. Vet Parasitol 70: 123–128. 9195716 10.1016/s0304-4017(96)01145-4

[42] Grice EA, Kong HH, Conlan S, Deming CB, Davis J, et al (2009) Topographical and temporal diversity of the human skin microbiome. Science 324: 1190–1192. 19478181 10.1126/science.1171700 PMC2805064

[43] Tran TV, Weisman LE (2004) Dexamethasone effects on group B streptococcal infection in newborn rats. Pediatr Infect Dis J 23: 47–52. 14743046 10.1097/01.inf.0000105107.76541.ee

[44] Hazarika RA, Mahanta PN, Dutta GN, Devriese LA (1991) Cutaneous infection associated with *Staphylococcus hyicus* in cattle. Res Vet Sci 50: 374–375. 1882147 10.1016/0034-5288(91)90146-f

[45] Andresen LO, Ahrens P, Daugaard L, Bille-Hansen V (2005) Exudative epidermitis in pigs caused by toxigenic *Staphylococcus chromogenes* . Vet Microbiol 105: 291–300. 15708827 10.1016/j.vetmic.2004.12.006

[46] Foster AP (2012) Staphylococcal skin disease in livestock. Vet Dermatol 23: : 342–351, e363. 10.1111/j.1365-3164.2012.01093.x 22823580

[47] Roberts S, Chambers S (2005) Diagnosis and management of *Staphylococcus aureus* infections of the skin and soft tissue. Intern Med J 35 Suppl 2S97–105. 16271065 10.1111/j.1444-0903.2005.00983.x

[48] Nagase N, Sasaki A, Yamashita K, Shimizu A, Wakita Y, et al (2002) Isolation and species distribution of staphylococci from animal and human skin. J Vet Med Sci 64: 245–250. 11999444 10.1292/jvms.64.245

[49] Capone KA, Dowd SE, Stamatas GN, Nikolovski J (2011) Diversity of the human skin microbiome early in life. J Invest Dermatol 131: 2026–2032. 21697884 10.1038/jid.2011.168 PMC3182836

[50] Hogg JC, Lehane MJ (1999) Identification of bacterial species associated with the sheep scab mite (*Psoroptes ovis*) by using amplified genes coding for 16S rRNA. Appl Environ Microbiol 65: 4227–4229. 10473440 10.1128/aem.65.9.4227-4229.1999 PMC99765

[51] Savini V, Catavitello C, Bianco A, Balbinot A, D'Antonio D (2009) Epidemiology, pathogenicity and emerging resistances in *Staphylococcus pasteuri*: from mammals and lampreys, to man. Recent Pat Antiinfect Drug Discov 4: 123–129. 19519547 10.2174/157489109788490352

[52] Rudolf I, Mendel J, Sikutová S, Svec P, Masaríková J, et al (2009) 16S rRNA gene-based identification of cultured bacterial flora from host-seeking *Ixodes ricinus*, *Dermacentor reticulatus* and *Haemaphysalis concinna* ticks, vectors of vertebrate pathogens. Folia Microbiol (Praha) 54: 419–428. 19937215 10.1007/s12223-009-0059-9

[53] Kloos WE, Schleifer KH (1983) *Staphylococcus auricularis* sp. *nov*.: an Inhabitant of the human external ear. International journal of systematic bacteriology 33 (1): 9–14 doi:10.1099/00207713-33-1-9

[54] Kluytmans JA (2010) Methicillin-resistant *Staphylococcus aureus* in food products: cause for concern or case for complacency? Clin Microbiol Infect 16: 11–15. 20002686 10.1111/j.1469-0691.2009.03110.x

[55] Carapetis JR, Connors C, Yarmirr D, Krause V, Currie BJ (1997) Success of a scabies control program in an Australian aboriginal community. Pediatr Infect Dis J 16: 494–499. 9154544 10.1097/00006454-199705000-00008

[56] Jackson A, Heukelbach J, Filho AF, Junior Ede B, Feldmeier H (2007) Clinical features and associated morbidity of scabies in a rural community in Alagoas, Brazil. Trop Med Int Health 12: 493–502. 17445140 10.1111/j.1365-3156.2006.01809.x

[57] Worth C, Heukelbach J, Fengler G, Walter B, Liesenfeld O, et al (2012) Acute morbidity associated with scabies and other ectoparasitoses rapidly improves after treatment with ivermectin. Pediatr Dermatol 29: 430–436. 22211573 10.1111/j.1525-1470.2011.01680.x

[58] Adjei O, Brenya RC (1997) Secondary bacterial infection in Ghanaian patients with scabies. East Afr Med J 74: 729–731. 9557448

[59] Mahe A (2001) Bacterial skin infections in a tropical environment. Curr Opin Infect Dis 14: 123–126. 11979120 10.1097/00001432-200104000-00002

[60] Lawrence G, Leafasia J, Sheridan J, Hills S, Wate J, et al (2005) Control of scabies, skin sores and haematuria in children in the Solomon Islands: another role for ivermectin. Bull World Health Organ 83: 34–42. 15682247 PMC2623469

[61] Otto M (2009) *Staphylococcus epidermidis*—the 'accidental' pathogen. Nat Rev Microbiol 7: 555–567. 19609257 10.1038/nrmicro2182 PMC2807625

[62] Duguid IG, Evans E, Brown MR, Gilbert P (1992) Growth-rate-independent killing by ciprofloxacin of biofilm-derived *Staphylococcus epidermidis*; evidence for cell-cycle dependency. J Antimicrob Chemother 30: 791–802. 1289353 10.1093/jac/30.6.791

[63] Foster TJ (2005) Immune evasion by staphylococci. Nat Rev Microbiol 3: 948–958. 16322743 10.1038/nrmicro1289

[64] Tong SY, McDonald MI, Holt DC, Currie BJ (2008) Global implications of the emergence of community-associated methicillin-resistant *Staphylococcus aureus* in Indigenous populations. Clin Infect Dis 46: 1871–1878. 18462175 10.1086/588301

[65] Devriese LA, Hommez J, Pot B, Haesebrouck F (1994) Identification and composition of the streptococcal and enterococcal flora of tonsils, intestines and faeces of pigs. J Appl Bacteriol 77: 31–36. 7928781 10.1111/j.1365-2672.1994.tb03040.x

[66] Hojberg O, Canibe N, Poulsen HD, Hedemann MS, Jensen BB (2005) Influence of dietary zinc oxide and copper sulfate on the gastrointestinal ecosystem in newly weaned piglets. Appl Environ Microbiol 71: 2267–2277. 15870311 10.1128/AEM.71.5.2267-2277.2005 PMC1087531

[67] Wertheim HF, Nghia HD, Taylor W, Schultsz C (2009) *Streptococcus suis*: an emerging human pathogen. Clin Infect Dis 48: 617–625. 19191650 10.1086/596763

[68] Gottschalk M, Xu J, Calzas C, Segura M (2010) *Streptococcus suis*: a new emerging or an old neglected zoonotic pathogen? Future Microbiol 5: 371–391. 20210549 10.2217/fmb.10.2

[69] Figueiro AR, Nunes ZG, Silvia AAL, Giordano-Dias CMG, Conra JR, et al.. (1995) Isolation of microorganisms of triatomines maintained in artificial and sylvatic conditions. Memorias do Instituto Oswaldo Cruz (Rio de Janeiro) 90.

[70] Durvasula RV, Sundaram RK, Kirsch P, Hurwitz I, Crawford CV, et al (2008) Genetic transformation of a Corynebacterial symbiont from the Chagas disease vector *Triatoma infestans* . Exp Parasitol 119: 94–98. 18331732 10.1016/j.exppara.2007.12.020 PMC2635415

