# Supplementary figures and images for: Scabies Mites Alter the Skin Microbiome and Promote Growth of Opportunistic Pathogens in a Porcine Model
Source: PLoS Negl Trop Dis. 2014 May 29;8(5):e2897. doi: 10.1371/journal.pntd.0002897 (PMC4038468; doi:10.1371/journal.pntd.0002897)

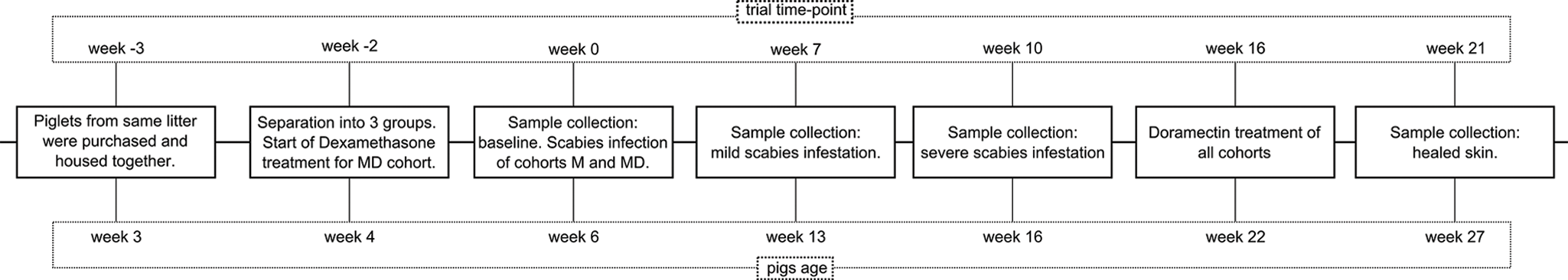

Supplement: Figure S1 — Timeline of experimental trial to monitor the scabies associated microbiota in the porcine model correlating relevant trial time points with the pigs' ages. (TIF) [file pntd.0002897.s001.tif]

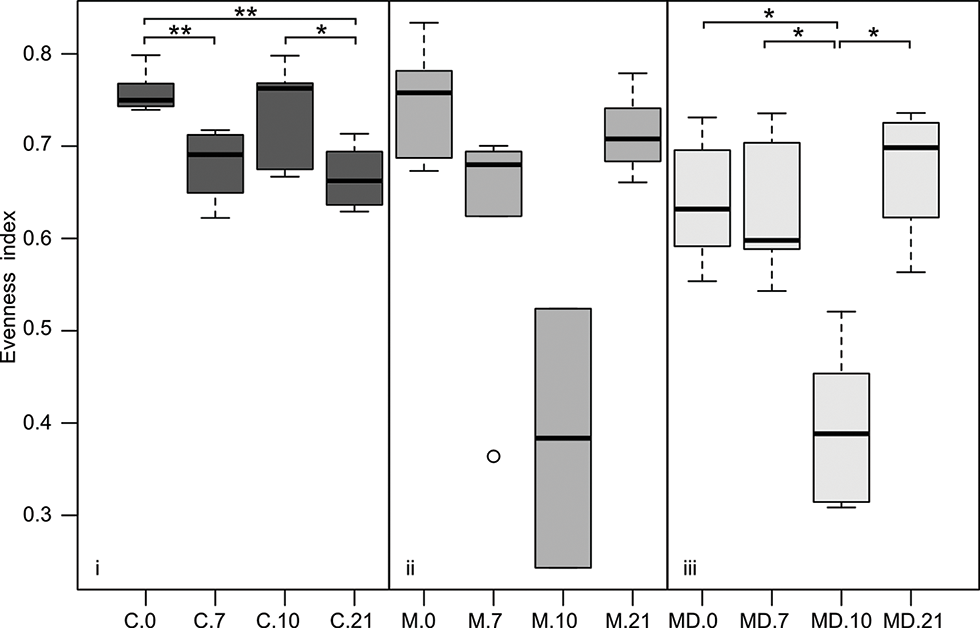

Supplement: Figure S2 — Effect of scabies mite infection on the evenness index for all samples from the cohorts C (i, mite free), M (ii, mite infested), MD (iii, mite infested and Dexamethasone treated). Evenness index is expressed as the Pielou's evenness (OTU level). Paired t-test was carried out for pigs where both time points were available (M.10: n = 2, MD: n = 4, other: n = 5). Significant differences are annotated by *: p<0.05, **:p<0.01, ***: p<0.001. (TIF) [file pntd.0002897.s002.tif]
